# Alcohol Use among Chinese Men Who Have Sex with Men: An Epidemiological Survey and Meta-Analysis

**DOI:** 10.1155/2014/414381

**Published:** 2014-03-11

**Authors:** Yu Liu, Han-Zhu Qian, Yuhua Ruan, Lu Yin, Juntao Ma, Kapil Dahiya, Wensheng Fan, Yiming Shao, Sten H. Vermund

**Affiliations:** ^1^Vanderbilt Institute for Global Health, Vanderbilt University, Nashville, TN 37203, USA; ^2^Division of Epidemiology, Department of Medicine, Vanderbilt University School of Medicine, Nashville, TN 37203, USA; ^3^State Key Laboratory for Infectious Disease Prevention and Control, National Center for AIDS/STD Control and Prevention, Chinese Center for Disease Control and Prevention, Collaborative Innovation Center for Diagnosis and Treatment of Infectious Diseases, Beijing 102206, China; ^4^Medical Library of Chinese People's Liberation Army, Beijing 100039, China; ^5^Department of Public Health, College of Health and Human Services, Western Kentucky University, Bowling Green, KY 42101, USA; ^6^Department of Pediatrics, Vanderbilt University School of Medicine, Nashville, TN 37203, USA

## Abstract

The HIV/AIDS epidemic among Chinese men who have sex with men (MSM) has become a significant public health concern. Knowledge of alcohol consumption in this population is limited. In this study, 1,155 Chinese MSM were surveyed to assess alcohol use and its correlates. A meta-analysis was also performed to aggregate pooled prevalence of current alcohol use. MSM who were unmarried (aOR: 1.87; 95% CI: 1.29–2.71) or unemployed/retired (aOR: 2.77; 95% CI: 1.73–4.45) were more likely to drink alcohol more than once per week. MSM who consumed alcohol more than once per week were more likely to use drug (*P* < 0.01), have sex with women (*P* < 0.01), have unprotected insertive (*P* = 0.04) or receptive (*P* = 0.03) anal sex with men, have more than 10 lifetime male sex partners (*P* < 0.01), predominantly practice insertive anal sex (*P* < 0.01), and trade sex for money (*P* < 0.01). Pooled overall alcohol use prevalence was 32%. Pooled prevalence for MSM who drank alcohol more than once per week and who drank alcohol before sex with male partners was 23%. Our findings provide the basis for further exploring the alcohol-HIV association and developing risk reduction interventions.

## 1. Introduction

HIV transmission is shifting in China from a preponderance of persons who inject drugs to those persons who acquire HIV sexually; homosexual transmission among men who have sex with men (MSM) is of particular concern [1]. The proportion of new HIV cases among Chinese MSM has surged from 0.2% in 2001 to 29.4% in 2011, and the major risk in this population is unprotected anal sex [2–4]. Prospective cohort studies have shown that Chinese MSM who experienced unprotected anal intercourse or had multiple male partners were 3–10 times more likely to encounter HIV seroconversion than MSM not practicing these risk behaviors [5–8].

Alcohol abuse is a factor in a variety of medical, social, and public health problems. Global evidence suggests that alcohol use is common among MSM, and can be associated with high risk behaviors for HIV and for other sexually transmitted infections (STI), such as unprotected sex, commercial sex, sexual violence, and sex with multiple concurrent partners [9–15].

Due to traditional social norms and booming economic development, alcohol is used commonly among Chinese men, especially those living in rural areas (26.4% among rural men versus 12.6% among urban men) [16]. Data from the China Chronic Disease and Risk Factor Surveillance study suggests that the prevalence of current drinking was 35.7%, higher among males (55.6%) than females (15.0%) [17]. Knowledge about prevalence of alcohol consumption prevalence and its important correlates among Chinese MSM is limited [18–20]. In this study, we sought to (1) evaluate the association of demographic factors with alcohol use among Chinese MSM; (2) compare risky behaviors among MSM who drank and who did not drink alcohol; and (3) perform a meta-analytic literature review to better estimate the prevalence of alcohol consumption among Chinese MSM with available published studies.

## 2. Materials and Methods

### 2.1. Study Design and Population

Our cross-sectional study was conducted among MSM in Beijing, China, during 2010 and 2011. The study design and study population were described in detail elsewhere [37]. In brief, the study participants were recruited from the community through a local gay-oriented community-based organization (CBO) and through referrals from ongoing epidemiological study. The primary objective of the study was to assess the relationship between male circumcision and HIV/HPV risks and a secondary objective was to explore alcohol use among MSM. Each study participant received genital exam on circumcision status, testing of blood and anal and genital swab specimens, and a questionnaire interview. Written informed consent was obtained. This study was approved by the institutional review boards of the National Center for AIDS/STD Control and Prevention of the Chinese Center for Disease Control and Prevention and Vanderbilt University School of Medicine.

### 2.2. Data Collection

A questionnaire interview collected data on sociodemographic characteristics (e.g., age, ethnicity, marital status, occupation, education, Beijing residence, and duration of living in Beijing), current alcohol drinking status (at least once per week), HIV risk behaviors (e.g., illicit drug use, sexual orientation, number of male or female sex partners and concurrent partners, specific sexual activities and condom use, anal sex role with male partners, commercial sex, and forced sex), and history of STI. Laboratory testing of HIV and syphilis was conducted according to Chinese national protocol.

For the meta-analysis on prevalence of current alcohol use among MSM in China, we searched both English and Chinese publications up to September 15, 2013, mainly in two databases: MEDLINE via PubMed and Wanfang Data, and we also searched Google Scholar for additional publications. The following search strategy was used to identify articles: (“China” OR “Chinese”) AND (“MSM” OR “men who have sex with men” OR “gay” OR “homosexual”) AND (“ethanol” OR “alcohol” OR “alcohols”). We also conduct reviews on the reference list of all eligible articles to identify additional literature relevant to the topic. Articles were considered eligible for inclusion if they were original epidemiologic studies conducted among MSM in China with reported alcohol use prevalence and/or had crucial statistics permitting us to compute prevalence. Two reviewers (Yu Liu and Han-Zhu Qian) examined relevant abstracts and determined whether they met criteria for inclusion.

### 2.3. Statistical Analysis

We sought to identify sociodemographic factors associated with alcohol consumption among Chinese MSM. First, we defined alcohol drinkers as someone who drank alcohol at least once per week in the past 4 weeks, and we compared sociodemographic factors between alcohol drinking MSM and nondrinkers in univariate logistic regression analysis. Significant factors were further evaluated by being fitting into multivariable logistic regression model. In each multivariable logistic model, potential confounders were adjusted* a priori *using direct acyclic graph (DAG). Odds ratios (OR) and 95% confidence intervals (CI) were calculated for both univariate and multivariable logistic regressions. We also compared risky behaviors between alcohol drinkers and nondrinkers by using Chi-squared test or a two-tailed Fisher's exact test.

In meta-analysis, we used three definitions on alcohol consumption: (1) the loose definition on drinking or not drinking alcohol which could mean different frequency and volume in the original papers; (2) ever drinking alcohol before sex; (3) drinking alcohol for at least once a week, which is consistent with that in our epidemiological survey. To account for the potential heterogeneity of studied population in each study reflected by different geographic locations, ethnicity, age group, and so forth, a random-effect model using the DerSimonian and Laird method [38, 39] was used to aggregate effect sizes to estimate the overall pooled prevalence and corresponding 95% CIs. Two separate random-effect models were also used to summarize the prevalence of alcohol consumption before sex with male sexual partners and alcohol use at least once per week. To quantify the heterogeneity of effect sizes over all included studies, *I* ^2^ statistics were calculated. Funnel plots were used to graphically examine signal of potential publication bias. Egger tests were performed to test the funnel plot asymmetry.

STATA 12.0 (StataCorp LP, College Station, Texas) was used for all statistical analyses.

## 3. Results

### 3.1. Demographics and Alcohol Use among MSM in Beijing

A total of 1,155 MSM provided informed consent to participate in the original study. Fifteen participants had missing information on alcohol drinking; therefore, 1140 (98.7%) participants were included in the analysis. Six percent were non-Han minority ethnics; 27% were married; 53% had ever attended college; 66% had Beijing residency; 59% had lived in Beijing for more than 4 years. Age ranged from 18 to 68 years (median = 30 years). The prevalence of drinking alcohol at least once a week was 23%.

### 3.2. Sociodemographic Factors Associated with Alcohol Consumption

Univariate analyses suggested the following factors to be associated with alcohol consumption: age, marriage, education, occupation, and sexual orientation. After controlling for age, education, and Beijing residency, being currently unmarried (adjusted OR [aOR]: 1.87; 95% CI: 1.29–2.71) and being unemployed/retired (aOR: 2.77; 95% CI: 1.73–4.45) were significantly associated with drinking alcohol at least once per week (Table 1).

### 3.3. Comparison of HIV Risk Behaviors between MSM Who Drank and Did Not Drink Alcohol

Alcohol drinking MSM were more likely to use illicit drug use (6.0% versus 1.9%; *P* < 0.01), to have female sexual partners in the past 6 months (61.5% versus 40.7%; *P* < 0.01), to have unprotected insertive (23.2% versus 17.6%; *P* = 0.04) or receptive (26.8% versus 18.3%; *P* = 0.03) anal sex with men in the past 6 months, to have ≥10 lifetime male sex partners (66.8% versus 59.8%; *P* = 0.04), to experience insertive anal sex with male partner in the past 6 months (80.4% versus 63.7%; *P* < 0.01), to predominantly practice insertive anal sex (52.7% versus 41.0%; *P* < 0.01), and to trade sex for money in the past 12 months (10.2% versus 3.5%; *P* < 0.01). MSM who drank alcohol were less likely to have more than one lifetime female sex partner (70.2% versus 76.2%; *P* = 0.04) and to be HIV (19.8% versus 27.3%; *P* = 0.02) infected or syphilis seropositive (21.2% versus 27.7%; *P* = 0.04) (Table 2).

### 3.4. Meta-Analysis of Literature on Alcohol Consumption among Chinese MSM

**Figure 1:**
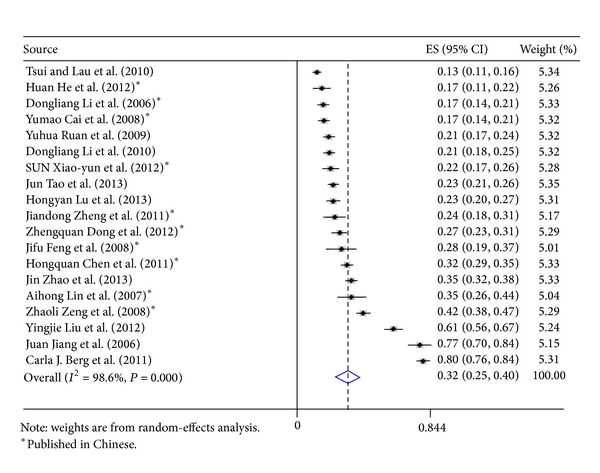
Forest plots of studies reporting prevalence of current alcohol consumption among Chinese men who have sex with men.

**Figure 2:**
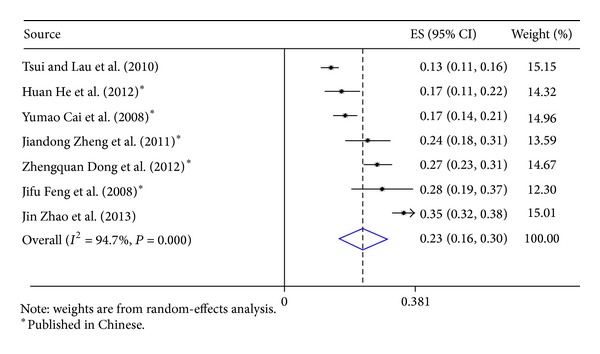
Forest plot of studies reporting prevalence of alcohol consumption before sex with male partners among Chinese men who have sex with men.

**Figure 3:**
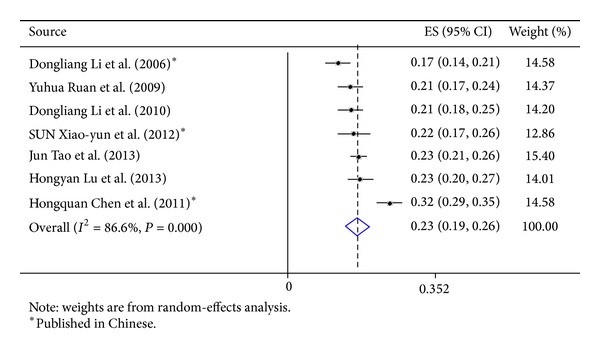
Forest plot of studies reporting prevalence of alcohol consumption ≥ once a week among Chinese men who have sex with men.

Our search strategy identified 2527 studies; 19 met the inclusion criteria and were included for computing the meta-analytic outcome of alcohol use prevalence (Table 3). All these 19 studies used cross-sectional design, and current alcohol use rates ranged from 16.5% to 79.7%; the pooled prevalence was 32% (effect size: 0.32; 95% CI: 0.25–0.40) (Figure 1). Five studies measured only general alcohol use status (yes versus no) [21, 24, 26, 31, 34]. Seven studies measured ever use of alcohol before sex [22, 23, 32, 35, 36], and the pooled prevalence was 23% (effect size: 0.23; 95% CI: 0.19–0.26) (Figure 2). Seven studies measured alcohol use at least once per week [8, 18, 19, 25, 30, 33], and the pooled prevalence was also 23% (effect size, 0.23; 95% CI, 0.19–0.26) (Figure 3). Large heterogeneity was observed among these studies (*I* ^2^ = 98.6%, *P* < 0.01). The funnel plot does not suggest significant publication bias (Egger's test *P* value = 0.13).

## 4. Discussion

In our survey of Chinese MSM living in Beijing, 23% of participants reported alcohol consumption at least once a week, and no association was noted with HIV or syphilis seropositivity. Unmarried and unemployed MSM were more likely to drink alcohol. Alcohol drinking MSM were more likely to report risky behaviors including illicit drug use, larger number of sexual partners, and unprotected sex. Alcohol has been consumed worldwide for a variety of recreational and psychological reasons. Because of its psychogenic nature and capability of impairing decision making [40], alcohol use has been associated with HIV/STI risk behaviors in both developed and developing countries [13, 14]. MSM may use alcohol for sexual arousal and expectation of casual sex [41]. Our findings are consistent with other studies that a higher frequency of alcohol consumption was associated with a greater likelihood of engaging in unprotected anal intercourse [2, 18, 19, 42, 43]. No association was seen with HIV or syphilis infections per se, suggesting transmission dynamics to be more complex that could be unraveled here.

Understanding of Chinese culture is particularly important in researching alcohol use among MSM [21]. Homosexual relationships are not legal in China and homosexuality is stigmatized in the general public because of traditional taboos and cultural discrimination [44]. Hence, Chinese MSM typically endure social isolation, stress, and low self-esteem; they suffer from psychological syndemics, as reported elsewhere in the world, representing multiple psychological vulnerabilities and related adverse health outcomes [9, 45]. With under one-quarter of Beijing MSM imbibing alcohol at least weekly, our survey found a lower alcohol consumption prevalence than the pooled prevalence among Chinese MSM that we calculated in our literature meta-analysis (32%; 95% CI: 25–40%). However, our findings are consistent with the geographic differences noted for alcohol consumption in the general Chinese population, since men in large cities are less likely to drink alcohol than in rural regions [16].

Our meta-analysis demonstrated that alcohol is often drunk before sex among Chinese MSM (pooled prevalence 23%), a well-known global risk factor for HIV and STIs. A longitudinal study in Rakai, Uganda, found the risk of contracting HIV among participant who used alcohol before sex was increased by 50% [46]. In China, about 30% of men who sought sex from female sex workers reported alcohol drinking before sex, and alcohol use was associated with less condom use and increased HIV/STIs in that context [47]. Since knowledge on factors associated with alcohol use before sex with male partners is scarce among Chinese MSM [48], more work is needed, particularly to assess casual alcohol use from heavy use, since it is the latter that is most commonly associated with high HIV/STI risks [49, 50]. Whether community-based health education may be helpful in raising consciousness about alcohol and higher risk taking among MSM is not known [51, 52].

There are several limitations in our study. First, alcohol consumption was based on self-reporting, and the data may be subjected to recall bias; since our study focused on circumcision, the alcohol use questions were not comprehensive—we did not measure the type and amount of alcohol that MSM consumed in the study [13, 53–58]. Second, the cross-sectional study design did not allow assessing the temporal relationship between alcohol consumption and HIV risk behaviors. Third, the study participants in the epidemiological survey were a convenience sample, including mixed sample from the community (HIV-unknown MSM) and from ongoing study (HIV-infected MSM), so no inference about true drinking prevalence can be justified, but the analysis on the risk factors for alcohol use provided useful information for developing intervention programs. Fourth, the studies included in the meta-analysis for computing pooled prevalence on alcohol drinking at least once per week were mostly conducted in Beijing; thus, the sample representativeness may limit the generalizability of our finding. Finally, there is high heterogeneity on alcohol use among included studies; it could be from within-study variance due to population characteristics and from between-study variances due to geographic variation and different metrics and mechanism of ascertaining alcohol use. However limited information is present to explore these factors and too few findings to warrant further stratified analyses. Despite these limitations, our study presents one of few large studies of investigating alcohol use and its correlates among MSM in China and ours is the first to assess alcohol use prevalence among Chinese MSM with a meta-analysis. Therefore, our study provides useful information for HIV prevention programs targeting this population and can signpost future work and intervention.

In summary, HIV and STI rates are rising among Chinese MSM and alcohol consumption is associated with increased risk-taking behaviors, likely through sexual disinhibition. To better understand the impact of alcohol consumption on sexual behaviors and HIV transmission among MSM, future studies should employ a prospective research design and more refined measures of alcohol use.

## Figures and Tables

**Table 1:** Sociodemographic predictors for alcohol drinking among men who have sex with men in Beijing, China (*N* = 1140).

Sociodemographic factors^a^	Alcohol consumption (*N*, %)		Crude OR (95% CI)	Adjusted OR (95% CI)^b^
	<once per week (*N* = 875)	≥once per week (*N* = 265)		
Age (year)			**1.03 (1.01, 1.05)**	1.00 (0.98, 1.02)
Median, IQR	30 (26–35)	32 (27–39)		
Ethnicity				
Han Chinese	821 (93.8)	252 (95.1)	Reference	—
Non-Han	54 (6.2)	13 (4.9)	0.78 (0.42, 1.46)	
Marital status				
Currently married	205 (23.4)	105 (39.6)	Reference	Reference
Currently unmarried	670 (76.6)	160 (60.4)	**2.14 (1.60, 2.87)**	**1.87 (1.29, 2.71)**
Education (years of schooling)				
College and above (>12)	480 (54.9)	122 (46.0)	Reference	
Senior high school (10–12)	240 (27.5)	85 (32.1)	**1.39 (1.01, 1.91)**	1.24 (0.90, 1.73)
Junior high school (7–9)	133 (15.2)	48 (18.1)	1.42 (0.97, 2.09)	1.21 (0.81, 1.80)
Primary school (≤6)	21 (2.4)	10 (3.8)	1.87 (0.86, 4.08)	1.43 (0.64, 3.21)
Occupation				
Employed	752 (86.0)	204 (77.0)	Reference	Reference
Unemployed/retired	47 (5.3)	34 (13.2)	**2.78 (1.76, 4.42)**	**2.77 (1.73, 4.45)**
Student	44 (5.1)	4 (1.5)	0.86 (0.44, 1.65)	0.84 (0.42, 1.77)
Other	32 (3.6)	22 (8.3)	1.52 (0.86, 2.73)	1.53 (0.90, 2.88)
Beijing residency				
No	292 (33.4)	94 (35.6)	Reference	—
Yes	583 (66.6)	170 (64.4)	0.91 (0.68, 1.21)	
Duration of living in Beijing (years)				
≤4	365 (41.7)	100 (37.7)	Reference	—
>4	510 (58.3)	165 (62.3)	1.18 (0.89, 1.57)	
Sexual orientation				
Homosexual	602 (69.8)	156 (59.3)	Reference	
Heterosexual	9 (1.0)	4 (1.5)	1.71 (0.52, 5.64)	1.60 (0.48, 5.33)
Bisexual	252 (29.2)	103 (39.2)	**1.58 (1.18, 2.11)**	1.28 (0.93, 1.75)

IQR: interquartile range; OR: odds ratio; CI: confidence interval.

^a^Sample size may vary due to missing data. ^b^Adjusted for age, education, and Beijing residency.

**Table 2:** Comparison of HIV risky behaviors between alcohol drinking and nondrinking men who have sex with men in Beijing, China (*N* = 1140).

Risk factors^a^	Alcohol consumption (*N*, %)		*P* value
	<once per week	≥once per week	
Illicit drug use			**<0.01**
No	856 (98.1)	249 (94.0)	
Yes	17 (1.9)	16 (6.0)	
Sexual orientation			**<0.01**
Homosexual	602 (69.8)	156 (59.3)	
Heterosexual	9 (1.0)	4 (1.5)	
Bisexual	252 (29.2)	103 (39.2)	
Sex with female partner in past 6 months			**<0.01**
No	519 (59.3)	102 (38.5)	
Yes	356 (40.7)	163 (61.5)	
Condom use with female partner in past 6 months			0.67
Every time	56 (27.5)	31 (30.4)	
Very often	20 (9.8)	6 (5.9)	
Rarely	29 (14.2)	16 (15.7)	
Never	99 (48.5)	49 (48)	
Number of lifetime female sex partners			**0.04**
<2	208 (23.8)	79 (29.8)	
≥2	667 (76.2)	186 (70.2)	
Oral sex with male partner in past 6 months			0.82
No	147 (16.8)	43 (16.2)	
Yes	726 (83.2)	222 (83.8)	
Condom use during oral sex with male partner in past 6 months			0.49
Every time	69 (9.5)	167 (75.2)	
Very often	36 (5.0)	28 (12.6)	
Rarely	72 (10.0)	7 (3.2)	
Never	546 (75.5)	20 (9.0)	
Insertive anal sex with male partner in past 6 months			**<0.01**
No	313 (36.3)	52 (19.6)	
Yes	549 (63.7)	213 (80.4)	
Condom use during insertive anal sex with male partner in past 6 months			**0.04**
Every time	324 (59.5)	117 (55.5)	
Very often	125 (22.9)	45 (21.3)	
Rarely	54 (9.9)	36 (17.0)	
Never	42 (7.7)	13 (6.2)	
Receptive anal sex with male partner in past 6 months			0.94
No	376 (43.6)	114 (43.3)	
Yes	486 (56.4)	149 (56.7)	
Condom use during receptive anal sex with male partner in past 6 months			**0.03**
Every time	296 (60.8)	74 (49.7)	
Very often	102 (20.9)	35 (23.5)	
Rarely	44 (9.1)	25 (16.8)	
Never	45 (9.2)	15 (10.0)	
Number of lifetime male sex partners			**<0.01**
<10	352 (40.2)	88 (33.2)	
≥10	523 (59.8)	177 (66.8)	
Anal sexual role with male partner			**<0.01**
Mainly/definitely receptive	299 (35.4)	59 (23.1)	
Mainly/definitely insertive	346 (41.0)	135 (52.7)	
Versatile^b^	199 (23.6)	62 (24.2)	
Multiple concurrent male partners in past 12 months			0.27
No	807 (93.0)	241 (90.9)	
Yes	61 (7.0)	24 (9.1)	
Traded sex for money in the past 12 months			**<0.01**
No	839 (96.5)	238 (89.8)	
Yes	30 (3.5)	27 (10.2)	
Forced sex with any male partner			0.08
No	843 (97.0)	251 (94.7)	
Yes	26 (3.0)	14 (5.3)	
Self-reported history of sexually transmitted diseases			0.84
No	588 (69.7)	180 (70.3)	
Yes	256 (30.3)	76 (29.7)	
Syphilis seropositive			**0.04**
No	633 (72.3)	208 (78.8)	
Yes	242 (27.7)	56 (21.2)	
HIV seropositive			**0.02**
No	635 (72.7)	211 (80.2)	
Yes	238 (27.3)	52 (19.8)	

^a^Sample size varies due to missing data.

^b^Versatile indicates mixed receptive and insertive anal sex roles.

**Table 3:** Summary of 19 quantitative studies on prevalence of alcohol use among Chinese men who have sex with men.

Source	City	Study design	Study participants		Alcohol measurement and time frame	Alcohol use prevalence (%) (number use alcohol/analytic sample)
			Total sample size	Age, median or mean; range		
Lu et al. (2013) [18]	Beijing	Cross-sectional	500	Median = 30; N/A	Alcohol use ≥ once per week (yes versus no) in the 12 months	23.4 (117/500)
Berg et al. (2011) [21]	Shanghai	Cross-sectional	404	N/A; N/A	General alcohol use (yes versus no) in the past 3 months	79.7 (322/404)
Li et al. (2010) [8]	Beijing	Cross-sectional	507	Median = 26; 18–62	Alcohol use ≥ once per week (yes versus no) in the past 3 months	21.1 (107/507)
Tsui and Lau (2010) [22]	Hong Kong	Cross-sectional	566	N/A; 18–60	Alcohol use before sex (yes versus no) in the past 12 months	13.3 (76/566)
Zhao et al. (2013) [23]	Shenzhen, Guangdong province	Cross-sectional	801	Median = 30; 18–62	Alcohol use before sex (yes versus no) in the past 12 months	34.8 (279/801)
Jiang et al. (2006) [24]	Five cities in Zhejiang province: Nanjing, Yangzhou, Suzhou, Wuxi, and Changzhou	Cross-sectional	137	N/A; 18–70	Ever used alcohol (yes versus no)	77.4 (106/137)
Tao et al. (2013) [25]	Beijing	Cross-sectional	1140	Median = 28; N/A	Alcohol use ≥ once per week (yes versus no) in the past 4 weeks	23.2 (265/1140)
Liu et al. (2012) [26]	Beijing	Cross-sectional	307	Mean = 23.7; N/A	Alcohol use (yes versus no) in the past 6 months	61.2 (188/307)
Ruan et al. (2009) [19]	Beijing	Cross-sectional	541	Median = 27; 18–62	Alcohol use ≥ once per week (yes versus no) in the past 3 months	20.7 (112/541)
Zheng et al. (2011) [27]	Beijing	Cross-sectional	157	Mean = 22.7; 17–32	Alcohol use before sex (yes versus no) in the past 6 months	24.2 (38/157)
Feng et al. (2008) [28]	Taizhou, Zhejiang province	Cross-sectional	95	Mean = 27.9; 18–48	Alcohol use before sex (yes versus no) in the past 1month	28.4 (27/95)
Chen et al. (2011) [29]	Nine cities: Harbin, Shenyang, Xi'an, Zhengzhou, Shanghai, Nanjing, Wuhan, Chongqing, and Chengdu	Cross-sectional	1470	N/A; N/A	Alcohol use ≥ once per week (yes versus no) in the past 12 months	32.0 (463/1447)
Li et al. (2006) [30]	Beijing	Cross-sectional	526	Median = 23; N/A	Alcohol use ≥ once per week (yes versus no) in the past 6 months	17.3 (91/526)
Lin et al. (2007) [31]	Shenzhen, Guangdong province	Cross-sectional	114	Mean = 30.5; 21–52	Ever used (yes versus no) alcohol	35.0 (40/114)
He et al. (2012) [32]	Shanghai	Cross-sectional	200	Mean = 36.3; 21–68	Alcohol use before sex (yes versus no) in the past 6 months	16.5 (33/200)
Xiao-yun et al. (2012) [33]	Beijing	Cross-sectional	304	Mean = 29.8; 18–49	Alcohol use ≥ once per week (yes versus no) in the past 4 weeks	21.7 (66/304)
Zeng et al. (2008) [34]	Beijing	Cross-sectional	541	Mean = 28.2; N/A	Alcohol use (yes versus no) in the past 3 months	42.1 (196/466)
Dong et al. (2012) [35]	Huzhou, Zhejiang province	Cross-sectional	410	Mean = 25.5; 15–47	Alcohol use before sex (yes versus no) in the past 6 months	27.1 (111/410)
Cai et al. (2008) [36]	Shenzhen, Guangdong province	Cross-sectional	458	Mean = 27.4; 18–53	Alcohol use before sex (yes versus no) in the past 12 months	17.5 (80/458)
